# Impact of INR monitoring, reversal agent use, heparin bridging, and anticoagulant interruption on rebleeding and thromboembolism in acute gastrointestinal bleeding

**DOI:** 10.1371/journal.pone.0183423

**Published:** 2017-09-01

**Authors:** Naoyoshi Nagata, Toshiyuki Sakurai, Shiori Moriyasu, Takuro Shimbo, Hidetaka Okubo, Kazuhiro Watanabe, Chizu Yokoi, Mikio Yanase, Junichi Akiyama, Naomi Uemura

**Affiliations:** 1 Department of Gastroenterology and Hepatology, National Center for Global Health and Medicine, Shinjuku, Tokyo, Japan; 2 Ohta Nishinouchi Hospital, Koriyama, Fukushima, Japan; 3 Department of Gastroenterology and Hepatology, Kohnodai Hospital, National Center for Global Health and Medicine, Ichikawa, Chiba, Japan; Ehime University Graduate School of Medicine, JAPAN

## Abstract

**Background:**

Anticoagulant management of acute gastrointestinal bleeding (GIB) during the pre-endoscopic period has not been fully addressed in American, European, or Asian guidelines. This study sought to evaluate the risks of rebleeding and thromboembolism in anticoagulated patients with acute GIB.

**Methods:**

Baseline, endoscopy, and outcome data were reviewed for 314 patients with acute GIB: 157 anticoagulant users and 157 age-, sex-, and important risk-matched non-users. Data were also compared between direct oral anticoagulants (DOACs) and warfarin users.

**Results:**

Between anticoagulant users and non-users, of whom 70% underwent early endoscopy, no endoscopy-related adverse events or significant differences were found in the rate of endoscopic therapy need, transfusion need, rebleeding, or thromboembolism. Rebleeding was associated with shock, comorbidities, low platelet count and albumin level, and low-dose aspirin use but not HAS-BLED score, any endoscopic results, heparin bridge, or international normalized ratio (INR) ≥ 2.5. Risks for thromboembolism were INR ≥ 2.5, difference in onset and pre-endoscopic INR, reversal agent use, and anticoagulant interruption but not CHA2DS2-VASc score, any endoscopic results, or heparin bridge. In patients without reversal agent use, heparin bridge, or anticoagulant interruption, there was only one rebleeding event and no thromboembolic events. Warfarin users had a significantly higher transfusion need than DOACs users.

**Conclusion:**

Endoscopy appears to be safe for anticoagulant users with acute GIB compared with non-users. Patient background factors were associated with rebleeding, whereas anticoagulant management factors (e.g. INR correction, reversal agent use, and drug interruption) were associated with thromboembolism. Early intervention without reversal agent use, heparin bridge, or anticoagulant interruption may be warranted for acute GIB.

## Introduction

Acute gastrointestinal bleeding (GIB) in patients who are taking oral anticoagulants is expected to increase as the population ages[1]. Endoscopy in this setting is a high-risk procedure[2–4] in that it is associated with the potential for rebleeding[4]. However, few data are available on the endoscopic and clinical outcomes of patients receiving anticoagulant therapy compared with those who are not[5,6].

The occurrence of acute GIB during anticoagulant therapy raises several difficulties related to the balance between bleeding risk and thromboembolic risk[2,3,7]. These risks are probably related to patient background, endoscopic results, and anticoagulant management[4,6,8,9]. However, which of these actually affect adverse outcomes remains unclear. Importantly, the issues of anticoagulant interruption, heparin bridge, and international normalized ratio (INR) correction during the pre-endoscopic period in the acute GIB setting have not been fully addressed in recent endoscopy guidelines from the United States, Europe, or Asia[2,3,10]. Many gastroenterologists, therefore, likely limit the management of anticoagulated patients to their own experiences.

Recently, direct oral anticoagulants (DOACs) have been approved as alternatives to warfarin[11]. While there may have been increasing recognition of GIB risk in patients on DOACs[11–13], limited data are available on differences in endoscopic results and adverse outcomes of GIB between DOAC and warfarin users[14].

The present study builds upon our previous work[15] by adding GIB cases who received oral anticoagulants and newly collecting detailed data on baseline, anticoagulant management, and endoscopic and rebleeding outcomes accordingly. Because only 2%-6% of patients with acute GIB use anticoagulants[16–18], we reviewed a large number of GIB patients to (i) determine whether anticoagulated patients with acute GIB have adverse endoscopic and clinical outcomes compared with those not receiving anticoagulants, (ii) elucidate the risk factors for rebleeding and thromboembolism following endoscopy in anticoagulated patients; and (iii) explore differences in the endoscopic and clinical outcomes of GIB between DOACs and warfarin users and between those with upper and lower GI.

## Material and methods

### Study design, setting, and participants

**Fig 1:**
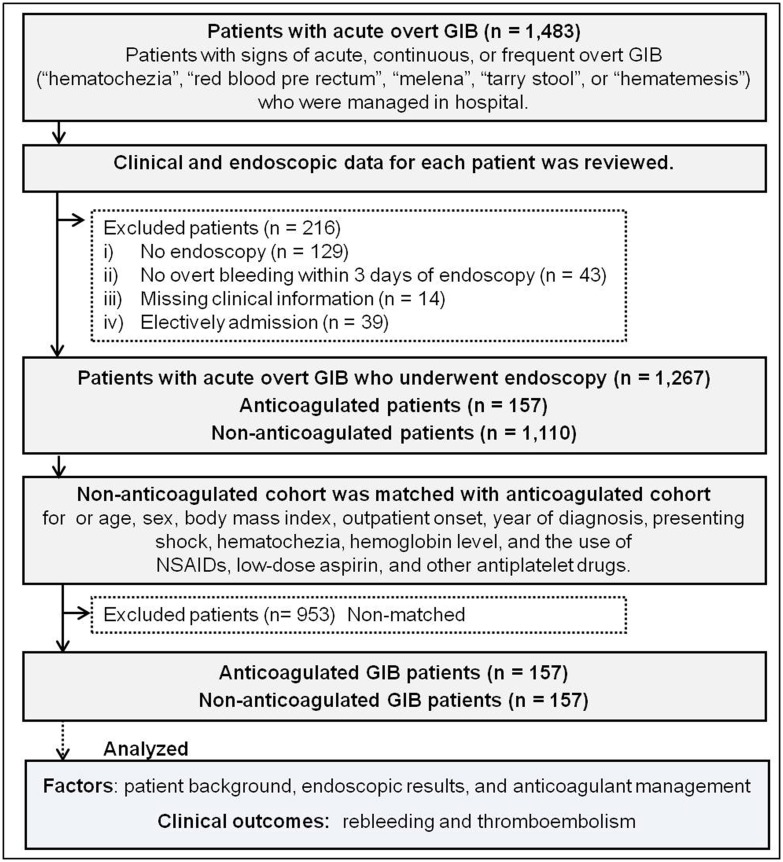
Study flow. Abbreviation. GIB, gastrointestinal bleeding.

We conducted a retrospective cohort study at the Department of Gastroenterology, National Center for Global Health and Medicine (NCGM), Japan. NCGM is the largest emergency hospital, with 900 beds, in the Tokyo metropolitan area. All clinical and endoscopic data analyzed were extracted from a prospective electronic medical database (MegaOak online imaging system, NEC, Japan) and an electronic endoscopic database (SolemioEndo, Olympus, Japan), both of which contain searchable collection of records into which physicians or nurses prospectively input all clinical findings immediately after clinical evaluation or endoscopy[19,20]. Fig 1 illustrates the study flow of patient selection.

Between January 2009 and May 2016, we identified 1,483 consecutive patients with signs of acute, continuous, or frequent overt GIB (“hematochezia”, “red blood per rectum”, “melena”, “tarry stool”, or “hematemesis”) who were managed in hospital. We then reviewed the clinical and endoscopic data for each patient and excluded those who: (1) did not undergo endoscopy (n = 129); (2) had no overt bleeding within 3 days of endoscopy (n = 43); (3) had some missing clinical information (n = 14); or (4) were electively admitted with GIB (n = 39). This left a cohort of 1,267 patients with acute GIB who underwent endoscopy from which we analyzed data for 157 anticoagulated patients and 157 non-anticoagulated patients matched for age, sex, body mass index, outpatient onset, year of diagnosis, presenting shock, hematochezia, hemoglobin level, and drug use (NSAIDs, low-dose aspirin [LDA], and other antiplatelets).

The requirement for patient consent was waived because this was a retrospective study that was conducted without invasive procedures and data were anonymized and deidentified by the director of the clinical research division at our institution, who was not involved in this study, before our analysis. This study was approved by the ethics committee of the National Center for Global Health and Medicine (No. 1579) and was conducted in accordance with the provisions of the Declaration of Helsinki.

### Comorbidities and medication

We collected data on the following background factors: signs of shock, presenting symptoms, history of GIB, co-morbidities, medications, and laboratory data related to GIB. Use of NSAIDs, LDA, non-aspirin antiplatelet drugs, warfarin, DOACs (dabigatran, rivaroxaban, apixaban and edoxaban), or proton pump inhibitors (PPIs) was defined as use within 1 month before admission. We evaluated 16 comorbidities using the Charlson comorbidity index[21]. CHA2DS2-VASc[22] and HAS-BLED[23] scores were calculated. After an episode of GIB, we collected data on anticoagulant management factors: INR in the pre-endoscopic period and use of reversal agent, heparin bridge, and drug interruption. Reversal agents included vitamin K, FFP, prothrombin complex concentrate (PCC), and recombinant activated factor VIIa. For heparin bridge, patients received prophylactic unfractionated heparin infusion intravenously. Only unfractionated heparin is used in Japan because low-molecular-weight heparin is not covered by the health care insurance system[24].

### Clinical outcomes

The main outcomes of interest were rebleeding and thromboembolism within 90 days of endoscopy. Rebleeding was defined as significant overt bleeding along with unstable vital signs, or the need for a transfusion of ≥ 2 units of packed red blood cells in the first 24 h after onset or endoscopically verified GIB. We defined a thromboembolic event as having acute coronary syndrome, stroke, transient ischemic attack, pulmonary embolism, deep vein thrombosis, or arterial thromboembolism. Workup for thromboembolism included positive computed tomography, magnetic resonance imaging, coronary angiography, ventilation-perfusion scanning, and/or ultrasonography.

### Statistics

Categorical data were compared between the groups using the Chi-square test or Fisher’s exact test, as appropriate. Continuous data were compared with Wilcoxon’s rank-sum test. To determine the predictive factors of clinical outcomes, we conducted univariate logistic regression analysis. Odds ratios (ORs) and 95% confidence intervals (CIs) were estimated. In terms of anticoagulant management factors, we developed 4 types of propensity score for DOAC use, PT-INR (onset) ≥ 2.5, reversal agent use, heparin bridge, and anticoagulant interruption by using a logistic regression model for each treatment that included background factors shown to be different (p<0.10) between treatment and non-treatment. Then, we conducted a multivariate analysis adjusted for the 4 types of propensity score to estimate the adjusted ORs for DOAC use, PT-INR (onset) ≥ 2.5, reversal agent use, heparin bridge, and anticoagulant interruption for clinical outcomes. A value of P < 0.05 was considered significant. All statistical analysis was performed using STATA version 14 software (StataCorp, College Station, TX).

## Results

### Background, endoscopy, and outcomes of anticoagulant users and non-users

The indications for anticoagulant use were as follows (Table 1): atrial fibrillation (n = 104), valvular replacement or valvular disease (n = 28), history of thromboembolism (n = 75); and ≥ 2 of these indications (n = 63). Baseline characteristics for both groups are shown in Table 1. The two groups were well matched for known risk factors, but compared with non-users, anticoagulated patients had significantly higher CHA2DS2-VASc scores, rates of atrial fibrillation and mechanical valve placement, prothrombin time (PT-INR) level, and use of PPIs. Endoscopic diagnosis or therapy is shown in Table 1: 108 patients (69%) underwent endoscopy within 24 h of onset in each group. Among anticoagulant users and non-users, 46.8% had upper sources and 53.2% had lower sources of GIB. The major cause of the bleeding was peptic ulcer disease for upper GIB and colonic diverticular bleeding for lower GIB. There were no significant differences between the two groups in terms of the rate of early endoscopy, upper or lower GIB, endoscopic diagnosis, endoscopic therapy need, transfusion need, rebleeding or thromboembolism. No endoscopy-related adverse events occurred in either group.

**Table 1:** Baseline characteristics and outcomes of GIB patients compared between anticoagulant users and matched non-user controls (n = 314).

**Characteristic**	**Anticoagulant users (n = 157)**	**Matched non-users (n = 157)**	**P value**
Age, years	75.7 ± 10.6	74.2 ± 11.5	0.198
Age > 70, years	118 (75.2)	117 (74.5)	0.310
Male	96 (61.2)	96 (61.2)	1.000
BMI ≥ 25, kg/m^2^	41 (26.1)	31 (19.8)	0.179
Inpatient onset	44 (28.0)	33 (21.0)	0.149
**Signs or symptoms**			
Shock^‡^	32 (20.4)	30 (19.1)	0.777
Hematemesis	25 (15.9)	34 (21.7)	0.194
Tarry stool	68 (43.3)	54 (34.4)	0.105
Hematochezia	84 (53.5)	84 (53.5)	1.000
**Comorbidities or past-history**			
Metabolic syndrome^†^	122 (77.7)	115 (73.3)	0.359
History of thromboembolism^††^	75 (47.8)	65 (41.4)	0.256
History of GI bleeding	34 (21.7)	27 (17.2)	0.318
Charlson comorbidity index	3.5 ± 2.7	3.9 ± 3.6	0.938
CHA2DS2-VASc score	2.7 ± 1.1	2.4 ± 1.1	**0.023**
HAS-BLED score	2.7 ± 1.2	2.5 ± 1.2	0.121
Atrial fibrillation	104 (66.2)	7 (4.5)	<**0.001**
Mechanical valve	21 (13.4)	0	<**0.001**
Biological valve	7 (4.5)	1 (0.6)	0.067
**Baseline laboratory data**			
Hemoglobin, g/dl	10.3 ± 4.6	9.7 ± 3.2	0.417
Platelets, 10^4^/μl	30.9 ± 148.8	20.7 ± 9.8	0.129
PT-INR	2.0 ± 1.2	1.1 ± 0.2	<**0.001**
PT-INR > 2.5	36 (22.9)	10 (6.4)	<**0.001**
Albumin, mg/dl	3.5 ± 2.4	3.1 ± 0.8	0.141
BUN, mg/dl	35.0 ± 22.4	31.9 ± 33.5	0.101
Creatinine, mg/dl	1.3 ± 1.3	1.7 ± 2.3	0.196
**Medications**			
NSAIDs	25 (15.9)	25 (15.9)	1.000
Low-dose aspirin	49 (31.2)	44 (28.0)	0.537
Thienopyridine^¶^	9 (5.7)	10 (6.4)	0.813
Other antiplatelets^¶^	10 (6.4)	6 (3.8)	0.305
Proton-pump inhibitors	78 (50.0)	41 (26.1)	<**0.001**
**Clinical outcome**	**Anticoagulant users (n = 157)**	**Matched non-users (n = 157)**	**P value**
Early endoscopy	108 (68.8)	108 (68.8)	1.00
Bleeding sources, lower GI tract	85 (54.1)	82 (52.2)	0.734
**Upper source**			
Peptic ulcer disease*	47 (29.9)	56 (35.7)	0.279
Mallory-Weiss syndrome	6 (3.8)	7 (4.5)	0.777
Post-endoscopic therapy	7 (4.5)	2 (1.3)	0.173
Esophageal ulcer	4 (2.6)	2 (1.3)	0.684
Angioectasia	2 (1.3)	2 (1.3)	1.000
Varices (esophagus or stomach)	0	3 (1.9)	0.248
Other diagnosis**	5 (3.2)	4 (2.6)	1.000
**Lower source**			
Colonic diverticular bleeding	26 (16.6)	29 (18.5)	0.656
Ischemic colitis	10 (6.4)	13 (8.3)	0.516
Other colitis	2 (1.3)	5 (3.2)	0.448
Colorectal cancer	5 (3.2)	3 (1.9)	0.723
Radiation proctitis	0	2 (1.3)	0.498
Angioectasia	7 (4.5)	1 (0.6)	0.067
Rectal ulcer	7 (4.5)	8 (5.1)	1.000
Inflammatory bowel disease	1 (0.6)	3 (1.9)	0.623
Post-endoscopic therapy	13 (8.3)	5 (3.2)	0.052
Hemorrhoids	4 (2.6)	4 (2.6)	1.000
Middle GIB	8 (5.1)	8 (5.1)	1.000
Other diagnosis***	2 (1.3)	0	0.498
Unknown****	1 (0.6)	1 (0.6)	1.000
**Endoscopic therapy need**	64 (40.8)	58 (36.9)	0.487
Clipping	55 (35.0)	47 (29.9)	0.335
Band ligation	5 (3.2)	9 (5.7)	0.413
Epinephrin injection therapy	3 (1.9)	3 (1.9)	1.000
Hemostatic forceps	3 (1.9)	0	0.248
Argon plasma coagulation	6 (3.8)	4 (2.6)	0.750
Interventional radiology need	0	1 (0.64)	1.000
Surgery need	1 (0.6)	2 (1.3)	1.000
Transfusion need	83 (52.9)	77 (49.0)	0.498
Units of Transfusion need	3.7 ± 5.3	4.5 ± 9.4	0.708
**Rebleeding**	21 (13.4)	25 (15.9)	0.523
**Thromboembolism**	9 (5.7)	5 (3.2)	0.677
Cardiovascular event	0	3 (1.9)	0.248
Cerebrovascular event	4 (2.6)	1 (0.6)	0.371
Pulmonary embolism or deep vein thrombosis^‡‡^	5 (3.2)	1 (0.6)	0.214

Values in parentheses are percentages. Values presented with a plus/minus sign are means ± SD. Bold values indicate statistical significance at P < 0.05.

^‡^Shock was defined as decrease in systolic blood pressure to < 90 mmHg, paleness, cold sweats, dizziness, syncope, or unconsciousness.

^†^Metabolic syndrome was a clustering of ≥ 2 of the 4 following medical conditions: abdominal (central) obesity, hypertension, diabetes mellitus, and dyslipidemia.

^††^History of thromboembolism was defined as the presence of acute coronary syndrome, stroke, transient ischemic attack, pulmonary embolism, deep vein thrombosis, or arterial thromboembolism.

^¶^Thienopyridine refers to the use of clopidogrel, prasugrel, ticagrelor, and ticlopidine. Other antiplatelets were cilostazol, dipyridamole, sarpogrelate hydrochloride, ethyl icosapentate, dilazep, limaprost, and beraprost.

^‡‡^Deep vein thrombosis occurred in 1 anticoagulant user and in 1 control.

*Peptic ulcer disease (n = 103) included gastric ulcer (n = 79) and duodenal ulcer (n = 26), and 2 patients had both gastric and duodenal ulcer. Five of the patients with gastric ulcer disease were subsequently identified as having gastric cancer based on histopathology.

**Other diagnosis of upper GIB included pancreatic cancer gastrointestinal invasion (n = 3), aneurysmal rupture to the stomach (n = 1), submucosal tumor of the stomach (n = 2), and bleeding from gastric polyp (n = 3).

***Other diagnosis of lower GIB bleeding was bleeding from colonic polyp (n = 2).

****Unknown source of bleeding (n = 2) was defined as a lesion where upper endoscopy and colonoscopy and/or capsule endoscopy or double-balloon endoscopy did not reveal the bleeding source.

Abbreviations: BMI, body mass index; PT-INR, prothrombin time-international normalized ratio; CHA2DS2-VASc, Congestive heart failure, Hypertension, Age ≥ 75, Diabetes mellitus, Stroke, Vascular disease, Sex female; HAS-BLED, hypertension, abnormal renal/liver function, stroke, bleeding history or predisposition, labile international normalized ratios (INR), elderly, drugs/alcohol concomitantly; BUN, blood urea nitrogen; NSAIDs, non-steroidal anti-inflammatory drugs.

### Risk factors for rebleeding and thromboembolism in anticoagulated patients

Factors associated with outcomes are shown in Tables 2–4. Significant predictors of rebleeding were found to be presenting shock, higher comorbidity index (including chronic kidney disease), platelet count < 10 (10^4^/μl), and low-dose aspirin use at admission but not HAS-BLED score (Table 2). No significant predictors of thromboembolic event were found. No associations were found between any of the endoscopic factors (endoscopy timing, bleeding site, etiology, and therapy) and any of the outcomes (Table 3). INR level at onset or pre-endoscopy did not predict rebleeding, but INR level at onset was a significant predictor of thromboembolism (Table 4). No significant difference in rebleeding rate was seen between INR < 2.5 and ≥ 2.5, but INR ≥ 2.5 at onset was a predictor of thromboembolism. The difference in INR value between onset and pre- or post-endoscopy was significantly associated with thromboembolism but not rebleeding. Reversal agent use was significantly associated with thromboembolism but not rebleeding. Heparin bridging was not significantly associated with rebleeding or thromboembolism. All thromboembolic events occurred in anticoagulant interrupted patients before endoscopy. In patients without reversal agent use, heparin bridge, or anticoagulant interruption, there was only one rebleeding event and no thromboembolic events.

**Table 2:** Patient background factors associated with rebleeding and thromboembolism in anticoagulant users with acute GI bleeding (n = 157).

**Factor**	**Rebleeding event/ no event**	**Odds ratio (95%CI)**	**P**	**Thromboembolic event/ no event**	**Odds ratio (95%CI)**	**P**
Age, years	77.1± 11.5/ 75.5 ± 10.4	1.0 (1.0–1.1)	0.507	70.7 ± 10.9/ 76.0 ± 10.5	0.9 (0.9–1.0)	0.148
Male	13 (61.9)/ 83 (61.0)	1.0 (0.4–2.7)	0.939	7 (77.8)/ 89 (60.1)	2.3 (0.5–11.6)	0.304
BMI ≥ 25, kg/m^2^	5 (23.8)/ 36 (26.5)	0.9 (0.3–2.5)	0.796	3 (33.3)/ 38 (25.7)	1.4 (0.3–6.1)	0.613
In-patient onset	6 (28.6)/ 38 (27.9)	1.0 (0.4–2.9)	0.952	2 (22.2)/ 42 (28.4)	0.7 (0.1–3.6)	0.691
Shock	8 (38.1)/ 24 (17.7)	2.9 (1.1–7.7)	**0.036**	3 (33.3)/ 29 (19.6)	2.1 (0.5–8.7)	0.329
Hematemesis	3 (14.3)/ 22 (16.2)	0.9 (0.2–3.2)	0.826	3 (33.3)/ 22 (14.9)	2.9 (0.7–12.3)	0.157
Tarry stool	8 (38.1)/ 60 (44.1)	0.8 (0.3–2.0)	0.605	5 (55.6)/ 63 (42.6)	1.7 (0.4–6.5)	0.449
Hematochezia	13 (61.9)/ 71 (52.2)	1.5 (0.6–3.8)	0.409	3 (33.3)/ 81 (54.7)	0.4 (0.1–1.7)	0.224
Metabolic syndrome^†^	16 (76.2)/ 106 (77.9)	0.9 (0.3–2.7)	0.858	6 (66.7)/ 106 (78.4)	0.6 (0.1–2.3)	0.418
History of thromboembolism^††^	12 (57.1)/ 63 (46.3)	1.5 (0.6–3.9)	0.358	6 (66.7)/ 69 (46.6)	2.3 (0.6–9.5)	0.254
History of GI bleeding	5 (23.8)/ 29 (21.3)	1.2 (0.4–3.4)	0.797	0/ 34 (23.0)	0.3 (0–1.8)*	0.208
Liver cirrhosis	1 (5.3)/ 4 (2.9)	1.9 (0.2–17.6)	0.588	0/ 5 (3.4)	NA^†^	NA^†^
Chronic kidney disease	3 (15.8)/ 5 (3.6)	5.0 (1.1–22.9)	**0.039**	1 (12.5)/ 7 (4.7)	2.9 (0.3–26.9)	0.349
Charlson comorbidity index	4.8 ± 3.0/ 3.3 ± 2.6	1.2 (1.0–1.4)	**0.024**	4.3 ± 4.3/ 3.5 ± 2.6	1.1 (0.9–1.4)	0.362
CHA2DS2-VASc	2.7 ± 0.8/ 2.7 ± 1.1	1.0 (0.7–1.5)	0.970	2.1 ± 0.9/ 2.7 ± 1.1	0.6 (0.3–1.1)	0.114
HAS-BLED score	3.0 ± 1.2/ 2.6 ± 1.2	1.4 (0.9–2.0)	0.135	2.7 ± 1.0/ 2.7 ± 1.2	1.0 (05–1.8)	0.941
Atrial fibrillation	12 (57.1)/ 92 (67.7)	0.6 (0.3–1.6)	0.346	5 (55.6)/ 99 (66.9)	0.6 (0.2–2.4)	0.489
Mechanical valve	3 (14.3)/ 18 (13.2)	1.1 (0.3–4.1)	0.895	2 (22.2)/ 19 (12.8)	1.9 (0.4–10.0)	0.429
**Factor**	**Rebleeding event/ no event**	**Odds ratio (95%CI)**	**P**	**Thromboembolic event/ no event**	**Odds ratio (95%CI)**	**P**
Biological valve	0/ 7 (5.2)	0.7 (0–4.6)*	0.717	0 / 7 (4.7)	1.7 (0–12.8)*	1.000
Hb < 7, g/dl	4 (19.1)/ 25 (18.4)	1.0 (0.3–3.4)	0.942	4 (44.4)/ 25 (16.9)	3.9 (0.9–15.7)	0.052
Platelets < 10, 10^4^/μl	6 (28.6)/ 10 (7.4)	5.0 (1.6–15.8)	**0.006**	0/ 16 (10.8)	0.7 (0–4.6)*	0.739
Alb < 3.0, mg/dl	10 (47.6)/ 35 (25.7)	2.6 (1.0–6.7)	**0.044**	5 (55.6)/ 40 (27.0)	3.4 (0.8–13.2)	0.080
BUN > 20.0, mg/dl	17 (81.0)/ 94 (69.1)	1.9 (0.6–6.0)	0.274	7 (77.8)/ 104 (70.3)	1.5 (0.3–7.4)	0.633
Creatinine >1.0, mg/dl	12 (57.1)/ 65 (47.8)	1.5 (0.6–3.7)	0.427	5 (55.6)/ 72 (48.7)	1.3 (0.3–5.1)	0.688
NSAID use (admission)	1 (4.8)/ 24 (17.7)	0.2 (0.03–1.8)	0.165	3 (33.3)/ 22 (14.9)	2.9 (0.7–12.3)	0.157
NSAID interruption	0/ 20 (83.3)	0.3 (0–9.8)*	0.400	2 (66.7)/ 18 (81.8)	0.4 (0.03–6.2)	0.546
LDA use (admission)	12 (52.4)/ 38 (27.9)	2.8 (1.1–7.2)	**0.029**	4 (44.4)/ 45 (30.4)	1.8 (0.5–7.1)	0.384
LDA interruption	10 (90.9)/ 32 (84.2)	1.9 (0.2–17.5)	0.581	4 (100)/ 38 (84.4)	0.9 (0.1-+inf)	1.000
Antiplatelet use (admission)	3 (14.3)/ 14 (10.3)	1.5 (0.4–5.6)	0.586	2 (22.2)/ 15 (10.1)	2.5 (0.5–13.3)	0.272
Antiplatelet interruption	3 (100)/ 13 (92.9)	0.2 (0.001-inf)*	1.000	2 (100)/ 14 (93.3)	0.1 (0.003-inf)*	1.000
PPI use (admission)	14 (66.7)/ 64 (47.1)	2.3 (0.9–5.9)	0.101	6 (66.7)/ 72 (48.7)	2.1 (0.5–8.8)	0.303
PPI continuation or start	6 (33.3)/ 29 (24.2)	1.6 (0.5–4.6)	0.407	1 (11.1)/ 34 (26.4)	0.3 (0.04–2.9)	0.330

Values in parentheses are percentages. Values presented with a plus/minus sign are means ± SD.

*Exact logistic regression was performed. Bold values mean statistical significance at P < 0.05.

^†^There were no patients with outcomes and statistical analysis was not performed.

^††^History of thromboembolism was defined as the presence of acute coronary syndrome, stroke, transient ischemic attack, pulmonary embolism, deep vein thrombosis, or arterial thromboembolism.

Abbreviations: BMI, body mass index; CHA2DS2-VASc, Congestive heart failure, Hypertension, Age ≥ 75, Diabetes mellitus, Stroke, Vascular disease, Sex female; HAS-BLED, hypertension, abnormal renal/liver function, stroke, bleeding history or predisposition, labile international normalized ratios (INR), elderly, drugs/alcohol concomitantly; BUN, blood urea nitrogen; NSAID, non-steroidal anti-inflammatory drugs; NA, not applicable; LDA, low-dose aspirin; PPI, proton-pump inhibitors; Inf, infinity.

**Table 3:** Endoscopic factors associated with rebleeding and thromboembolism in anticoagulant users with acute GI bleeding (n = 157).

Factor	Rebleeding event/ no event	Odds ratio (95%CI)	P	Thromboembolic event/ no event	Odds ratio (95%CI)	P
Early endoscopy (≤ 24 h)	13 (61.9)/ 90 (69.9)	0.7 (0.3–1.8)	0.466	5 (55.6)/ 103 (69.6)	0.5 (0.1–2.1)	0.384
Lower GIB vs upper GIB	12 (57.1)/ 73 (53.7)	1.2 (0.5–2.9)	0.767	3 (33.3)/ 82 (55.4)	0.4 (0.1–1.7)	0.210
Peptic ulcer bleeding	4 (19.1)/ 43 (31.6)	0.5 (0.2–1.6)	0.249	4 (44.4)/ 43 (29.1)	2.0 (05–7.6)	0.335
Colonic diverticular bleeding	3 (14.3)/ 23 (16.9)	0.8 (0.2–3.0)	0.763	0/ 26 (17.6)	0.4 (0.01–2.4)*	0.523
Received endoscopic therapy	11 (52.4)/ 53 (39.0)	1.7 (0.7–4.3)	0.248	4 (44.4)/ 60 (40.5)	1.2 (0.3–4.5)	0.817
Received endoscopic clipping	8 (38.1)/ 47 (34.6)	1.2 (0.5–3.0)	0.752	3 (33.3)/ 52 (35.1)	0.9 (0.2–3.8)	0.912
Received endoscopic ligation	1 (4.8)/ 4 (2.9)	1.7 (0.2–15.5)	0.661	0/ 5 (3.4)	1.7 (0.04–13.1)*	0.959

Values in parentheses are percentages. Values presented with a plus/minus sign are means ± SD.

*Exact logistic regression was performed. Bold values indicate statistical significance at P < 0.05.

Abbreviation. GIB, gastrointestinal bleeding.

**Table 4:** Anticoagulant management factors associated with rebleeding and thromboembolism in anticoagulant users with acute GI bleeding (n = 157).

Factor	Rebleeding event/ no event	Odds ratio (95%CI)	P	Thromboembolic event/ no event	Odds ratio (95%CI)	P
DOAC use	5 (23.8)/ 37 (27.2)	0.8 (0.3–2.4)	0.744	1 (11.1)/ 41 (27.1)	0.3 (0.04–2.7)	0.298
PT-INR (onset)	1.8 ± 0.8/ 1.9 ± 0.8	0.8 (0.4–1.5)	0.517	2.6 ± 1.0/ 1.8 ± 0.7	3.1 (1.4–7.2)	**0.008**
PT-INR (pre-endoscopy)	1.6 ± 0.7/ 1.7 ± 0.7	0.8 (0.4–1.6)	0.568	2.1 ± 1.0/ 1.7 ± 0.7	1.8 (0.8–4.1)	0.139
PT-INR (onset) ≥ 2.5	4 (19.1)/ 32 (23.5)	0.8 (0.2–2.4)	0.650	6 (66.7)/ 30 (20.3)	7.9 (1.9–33.3)	**0.005**
PT-INR (pre-endoscopy) ≥ 2.5	2 (9.5)/ 25 (18.4)	0.5 (0.1–2.1)	0.327	3 (33.3)/ 24 (16.2)	2.6 (0.6–11.0)	0.201
Difference of INR value between admission and pre-endoscopy	0.2 ± 0.3/ 0.2 ± 0.4	0.9 (0.3–3.1)	0.974	0.5 ± 0.8/ 0.2 ± 0.4	3.7 (1.2–11.3)	**0.022**
Difference of INR value between onset and post-endoscopy	0.5 ± 0.8/ 0.5 ± 0.7	1.0 (0.5–2.0)	0.897	1.3 ± 1.0/ 0.4 ± 0.7	3.2 (1.5–6.7)	**0.002**
Reversal agent (Vitamin K antagonist) use^¶^	4 (19.1)/ 24 (17.7)	1.1 (0.3–3.6)	0.876	4 (44.4)/ 24 (16.2)	4.1 (1.0–16.5)	**0.045**
Heparin bridging	7 (33.3)/ 42 (30.9)	1.1 (0.4–3.0)	0.822	4 (44.4)/ 45 (30.4)	1.8 (0.5–7.1)	0.384
Anticoagulant interruption before endoscopy	19 (90.5)/ 97 (71.3)	3.8 (0.8–17.2)	0.081	9 (100)/ 107 (72.3)	4.7 (0.7-inf)*	0.121
No reversal agent use, no heparin bridge, or no anticoagulant interruption	1 (4.8)/ 36 (26.5)	0.1 (0.02–1.1)	0.058	0/ 37 (25)	0.2 (0–1.6)*	0.165

Values in parentheses are percentages. Values presented with a plus/minus sign are means ± SD.

*Exact logistic regression was performed.

Bold values denote statistical significance at P < 0.05.

^¶^Twenty-eight of patients received a reversal agent (Vitamin K) intravenously during the peri-endoscopic period; no patients received FFP, prothrombin complex concentrate, or recombinant activated factor VIIa.

Abbreviations: PT-INR, prothrombin time-international normalized ratio; Inf, infinity.

Even after propensity score adjustment, the risk of DOAC use, PT-INR ≥ 2.5 at onset, heparin bridge, or anticoagulant interruption for any of the clinical outcomes remained unchanged compared with univariate analysis. Reversal agent use was not significantly associated with thromboembolism after propensity score adjustment (Table 5).

**Table 5:** Anticoagulant management factors associated with rebleeding and thromboembolism in GI bleeders after propensity score adjustment (n = 157).

	Propensity score for each treatment	Rebleeding risk		Thromboembolic risk	
	C-statistic (95%CI)	Adjusted odds ratio (95%CI)	P	Adjusted odds ratio (95%CI)	P
DOAC use	0.81 (0.74–0.88)	0.8 (0.2–2.5)	0.648	0.6 (0.06–5.9)	0.646
PT-INR (onset) ≥ 2.5	0.74 (0.65–0.83)	0.7 (0.2–2.3)	0.523	7.3 (1.5–35.3)	**0.013**
Reversal agent (vitamin K antagonist) use	0.92 (0.87–0.97)	1.4 (0.3–7.3)	0.722	1.2 (0.2–9.1)	0.840
Heparin bridging	0.77 (0.68–0.85)	0.8 (0.3–2.3)	0.648	1.3 (0.3–5.8)	0.767
Anticoagulant interruption before endoscopy	0.79 (0.71–0.86)	3.3 (0.7–16.1)	0.081	NA^†^	NA^†^

Bold values denote statistical significance at P < 0.05.

To estimate propensity scores, the following logistic regression models were used. For DOAC use, the model included 8 factors shown to be different on univariate analysis (P < 0.10) between DOAC use and non-use: being elderly (age ≥ 70 years), sex, past history of thromboembolism, CHA2DS2-VASc score, atrial fibrillation, hemoglobin, INR > 2.5, and low-dose aspirin use.

For INR > 2.5, the model included 9 factors different on univariate analysis (P < 0.10) between INR > 2.5 use and INR ≤ 2.5: being elderly (age ≥ 70 years), sex, past history of thromboembolism, past history of gastrointestinal bleeding, HAS-BLED score, atrial fibrillation, mechanical valve, biological valve, and DOAC use.

For reversal agent (vitamin K antagonist) use, the model included 11 factors different on univariate analysis (P < 0.10) between reversal agent use and non-use: being elderly (age ≥ 70 years), sex, hematemesis, metabolic syndrome, Charlson comorbidity index score, CHA2DS2-VASc score, atrial fibrillation, mechanical valve, hemoglobin, INR > 2.5, and DOAC use.

For heparin bridge, the model included 6 factors different on univariate analysis (P < 0.10) between heparin bridge use and non-heparin bridge: being elderly (age ≥ 70 years), sex, NSAID use, low-dose aspirin use, non-aspirin antiplatelet use, and DOAC use.

For anticoagulant interruption, the model included 9 factors different on univariate analysis (P < 0.10) between heparin bridge use and non-heparin bridge: being elderly (age ≥ 70 years), sex, BMI ≥ 25, inpatient onset, past history of gastrointestinal bleeding, INR > 2.5, low-dose aspirin use, non-aspirin antiplatelet use, and DOAC use.

^†^All interrupted patients had thromboembolism and statistical analysis could not be performed.

Abbreviations: PT-INR, prothrombin time-international normalized ratio; DOAC, direct oral anticoagulant.

### Subgroup analysis of DOAC and warfarin users

Compared with warfarin users, DOAC users had a significantly higher rate of atrial fibrillation, higher levels of hemoglobin and albumin, lower levels of PT-INR and BUN, a lower rate of LDA use, and a higher rate of lower GIB (Table 6). No significant difference was found in the rate of early endoscopy, other endoscopic diagnosis, or other endoscopic therapy between the groups (Table 6). In terms of clinical outcomes, DOAC users received significantly fewer transfusions than warfarin users, and no significant differences were found in endoscopic therapy need, rebleeding, or thromboembolism between the groups (Table 6).

**Table 6:** Baseline characteristics and outcomes of GIB compared between direct oral anticoagulant (DOAC) and warfarin users (n = 157).

**Characteristic**	**DOAC users (n = 42)**	**Warfarin users (n = 115)**	**P value**
Age, years	77.6 ± 7.7	75.0 ± 11.4	0.385
Age > 70, years	34 (81.0)	84 (73.0)	0.310
Male	23 (54.8)	73 (63.4)	0.321
BMI ≥ 25, kg/m^2^	13 (31.0)	28 (24.4)	0.404
Inpatient onset	11 (26.2)	33 (28.7)	0.757
**Signs or symptoms**			
Shock	6 (14.3)	26 (22.6)	0.252
Hematemesis	4 (9.5)	21 (18.3)	0.185
Tarry stool	19 (45.2)	49 (42.6)	0.769
Hematochezia	25 (59.5)	59 (51.3)	0.361
**Comorbidities or past-history**			
Metabolic syndrome^†^	31 (73.8)	91 (79.1)	0.478
History of thromboembolism^††^	25 (59.5)	50 (43.5)	0.075
History of GI bleeding	11 (26.2)	23 (20.0)	0.405
Charlson comorbidity index	3.4 ± 3.0	3.6 ± 2.6	0.315
CHA2DS2-VASc score	2.9 ± 0.9	2.6 ± 1.1	0.174
HAS-BLED score	2.8 ± 1.0	2.7 ± 1.2	0.635
Atrial fibrillation	34 (81.0)	70 (60.9)	**0.018**
Mechanical valve	5 (11.9)	16 (13.9)	0.743
Biological valve	2 (4.8)	5 (4.4)	1.000
**Baseline laboratory data**			
Hemoglobin, g/dl	10.9 ± 2.9	10.1 ± 5.1	**0.030**
Platelets, 10^4^/μl	18.4 ± 6.8	35.5 ± 173.7	0.827
PT-INR	1.3 ± 0.3	2.2 ± 1.3	<**0.001**
PT-INR > 2.5	3 (7.1)	33 (28.7)	**0.005**
Albumin, mg/dl	3.5 ± 0.6	3.4 ± 2.8	**0.003**
BUN, mg/dl	30.2 ± 27.1	36.7 ± 21.8	**0.001**
Creatinine, mg/dl	1.0 ± 0.4	1.4 ± 1.4	0.400
**Medications**			
NSAIDs	6 (14.3)	19 (16.5)	0.735
Low-dose aspirin	8 (19.1)	41 (35.7)	**0.047**
Thienopyridine	2 (4.7)	7 (6.1)	1.000
Other antiplatelets^¶^	1 (2.4	9 (7.8)	0.291
Proton-pump inhibitors	23 (54.8)	55 (47.8)	0.442
**Clinical outcome**	**DOAC users (n = 42)**	**Warfarin users (n = 115)**	**P value**
Early endoscopy	31 (73.8)	77 (67)	0.412
Bleeding sources, lower GI tract	29 (69.1)	56 (48.7)	**0.023**
**Upper source**			
Peptic ulcer disease*	9 (21.4)	38 (33.0)	0.160
Mallory-Weiss syndrome	2 (4.8)	5 (4.4)	1.000
Post-endoscopic therapy	0	7 (6.1)	0.191
Esophageal ulcer	3 (2.6)	1 (0.9)	1.000
Angioectasia	0	2 (1.7)	1.000
Varices (esophagus or stomach)	0	0	NA
Other diagnosis**	2 (4.8)	3 (2.6)	0.610
**Lower source**			
Colonic diverticular bleeding	8 (19.1)	18 (15.7)	0.612
Ischemic colitis	2 (4.8)	8 (7.0)	1.000
Other colitis	0	2 (1.7)	1.000
Colorectal cancer	0	5 (4.4)	0.325
Radiation proctitis	0	0	NA
Angioectasia	3 (7.1)	4 (3.5)	0.385
Rectal ulcer	1 (2.4)	6 (5.2)	0.676
Inflammatory bowel disease	0	1 (0.9)	1.000
Post-endoscopic therapy	8 (19.1)	5 (4.4)	**0.006**
Hemorrhoids	2 (4.8)	2 (1.7)	0.290
Middle GIB	5 (11.9)	3 (2.6)	**0.032**
Other diagnosis***	0	2 (1.7)	1.000
Unknown****	0	1 (0.9)	1.000
**Endoscopic therapy need**	18 (42.9)	46 (40.0)	0.747
Clipping	14 (33.3)	41 (35.7)	0.787
Band ligation	4 (9.5)	1 (0.9)	**0.018**
Epinephrin injection therapy	0	2 (6.1)	0.565
Hemostatic forceps	0	3 (2.6)	0.565
Argon plasma coagulation	0	6 (5.2)	0.193
Combined therapy	0	6 (5.2)	0.193
Interventional radiology need	0	0	NA
Surgery need	0	1 (0.9)	1.000
Transfusion need	17 (40.5)	66 (57.4)	0.072
Units of transfusion needed	2.2 ± 3.1	4.3 ± 5.9	**0.046**
**Rebleeding**	5 (11.9)	16 (13.9)	0.743
**Thromboembolism**	1 (2.4)	8 (7.0)	0.446

Values in parentheses are percentages. Values presented with a plus/minus sign are means ± SD. Bold values indicate statistical significance at P < 0.05.

^†^Metabolic syndrome was a clustering of at least two of the four following medical conditions: abdominal (central) obesity, hypertension, diabetes mellitus, and dyslipidemia.

^††^History of thromboembolism was defined as the presence of acute coronary syndrome, stroke, transient ischemic attack, pulmonary embolism, deep vein thrombosis, or arterial thromboembolism.

^¶^Other antiplatelets were cilostazol, dipyridamole, sarpogrelate hydrochloride, ethyl icosapentate, dilazep, limaprost, and beraprost.

*Peptic ulcer disease (n = 103) included gastric ulcer (n = 79) and duodenal ulcer (n = 26), and 2 patients had both gastric and duodenal ulcer. Five of the patients with gastric ulcer disease were subsequently identified as having gastric cancer based on histopathology.

**Other diagnosis of upper GIB included pancreatic cancer gastrointestinal invasion (n = 3), aneurysmal rupture to the stomach (n = 1), submucosal tumor of the stomach (n = 2), and bleeding from gastric polyp (n = 3).

***Other diagnosis of lower GIB bleeding was bleeding from colonic polyp (n = 2).

****Unknown source of bleeding (n = 2) was defined as a lesion where upper endoscopy and colonoscopy and/or capsule endoscopy or double-balloon endoscopy did not reveal the bleeding source.

Abbreviations: BMI, body mass index; PT-INR, prothrombin time-international normalized ratio; CHA2DS2-VASc, Congestive heart failure, Hypertension, Age ≥ 75, Diabetes mellitus, Stroke, Vascular disease, Sex female; DOAC, direct oral anticoagulant; HAS-BLED, hypertension, abnormal renal/liver function, stroke, bleeding history or predisposition, labile international normalized ratios (INR), elderly, drugs/alcohol concomitantly; BUN, blood urea nitrogen; NSAIDs, non-steroidal anti-inflammatory drugs.

### Subgroup analysis of patients diagnosed with upper and lower GIB

Compared with lower GI bleeders, there was a significantly higher rate of upper GI bleeders in elderly patients (> 70 years) and patients with shock, hematemesis, tarry stool, lower hemoglobin and albumin levels, or higher BUN level (Table 7). Upper GI bleeders were associated with a significantly higher rate of early endoscopy, endoscopic therapy need (particularly clipping), and transfusion need (Table 7).

**Table 7:** Baseline characteristics and outcomes of anticoagulant users compared between upper and lower GI bleeding (n = 157).

**Characteristic**	**Upper GIB (n = 72)**	**Lower GIB (n = 85)**	P value
Age, years	47 (65.3)	71 (83.5)	**0.008**
Age > 70, years	73.9±11.2	77.2±9.8	**0.046**
Male	48 (56.5)	48 (66.7)	0.192
BMI ≥ 25, kg/m^2^	21 (24.7)	20 (27.8)	0.662
Inpatient onset	19 (22.4)	25 (34.7)	0.086
**Signs or symptoms**			
Shock^‡^	20 (27.8)	12 (14.1)	**0.034**
Hematemesis	23 (31.9)	2 (2.4)	<**0.001**
Tarry stool	54 (75.0)	14 (16.5)	<**0.001**
Hematochezia	8 (11.1)	76 (89.4)	<**0.001**
**Comorbidities or past history**			
Metabolic syndrome^†^	54 (75.0)	68 (80.0)	0.453
History of thromboembolism^††^	33 (45.8)	42 (49.4)	0.655
History of GI bleeding	13 (18.1)	21 (24.7)	0.313
Charlson comorbidity index	55 (76.4)	59 (69.4)	0.329
CHA2DS2-VASc score	62 (86.1)	78 (91.8)	0.256
HAS-BLED score	39 (54.2)	49 (57.7)	0.662
Atrial fibrillation	47 (65.3)	57 (67.1)	0.814
Mechanical valve	8 (11.1)	13 (15.3)	0.443
Biological valve	3 (4.2)	4 (4.7)	1.000
**Baseline laboratory data**			
Hemoglobin, g/dl	8.8±2.9	11.5±5.4	<**0.001**
Platelets, 10^4^/μl	20.0±9.0	40.1±202.1	0.224
PT-INR	2.1±1.3	1.9±1.1	0.140
PT-INR > 2.5	21 (29.2)	15 (17.7)	0.087
Albumin, mg/dl	3.1±0.6	3.8±3.2	<**0.001**
BUN, mg/dl	44.9±25.1	26.6±18.1	<**0.001**
Creatinine, mg/dl	1.4±1.5	1.2±1.0	0.948
**Medications**			
NSAIDs	14 (19.4)	11 (12.9)	0.267
LDA	27 (37.5)	22 (25.9)	0.117
Thienopyridine	3 (4.2)	6 (7.1)	0.509
Other antiplatelets^¶^	4 (5.6)	6 (7.1)	0.755
PPIs	32 (44.4)	46 (54.1)	0.227
**Clinical outcomes**	**Upper GIB (n = 72)**	**Lower GIB (n = 85)**	P value
Early endoscopy	60 (83.3)	48 (56.5)	<**0.001**
**Endoscopic therapy need**	37 (51.4)	27 (31.8)	**0.013**
Clipping	34 (47.2)	21 (24.7)	**0.003**
Band ligation	1 (1.4)	4 (4.7)	0.376
Epinephrin injection therapy	3 (4.2)	0	0.094
Hemostatic forceps	1 (1.4)	2 (2.4)	1.000
Argon plasma coagulation	2 (2.8)	4 (4.7)	0.688
Combined therapy	4 (5.6)	2 (2.4)	0.414
Interventional radiology need	0	0	NA
Surgery need	0	1 (1.2)	1.000
Transfusion need	51 (70.8)	32 (37.7)	<**0.001**
Units of Transfusion need			
**Rebleeding**	9 (12.5)	12 (14.1)	0.767
**Thromboembolism**	6 (8.3)	3 (3.5)	0.303

Values in parentheses are percentages. Values presented with a plus/minus sign are means ± SD. Bold values mean statistical significance at P < 0.05.

^‡^Shock was defined as decrease in systolic blood pressure to < 90 mmHg, paleness, cold sweats, dizziness, syncope, or unconsciousness.

^†^Metabolic syndrome was a clustering of ≥ 2 of the 4 following medical conditions: abdominal (central) obesity, hypertension, diabetes mellitus, and dyslipidemia.

^††^History of thromboembolism was defined as the presence of acute coronary syndrome, stroke, transient ischemic attack, pulmonary embolism, deep vein thrombosis, or arterial thromboembolism.

^¶^Other antiplatelets were cilostazol, dipyridamole, sarpogrelate hydrochloride, ethyl icosapentate, dilazep, limaprost, and beraprost.

Abbreviations: BMI, body mass index; PT-INR, prothrombin time-international normalized ratio; CHA2DS2-VASc, Congestive heart failure, Hypertension, Age ≥ 75, Diabetes mellitus, Stroke, Vascular disease, Sex female; HAS-BLED, hypertension, abnormal renal/liver function, stroke, bleeding history or predisposition, labile international normalized ratios [INR], elderly, drugs/alcohol concomitantly; BUN, blood urea nitrogen; NSAIDs, non-steroidal anti-inflammatory drugs; LDA, low-dose aspirin; PPIs, Proton-pump inhibitors.

## Discussion

This study presents new information on the anticoagulant management for acute GIB. First, there were no apparent differences in endoscopic results or adverse clinical outcomes of GIB between anticoagulant users and non-anticoagulant users matched for age, sex, and important risk factors as controls. Second, only patient background factors were associated with rebleeding, whereas only anticoagulant management factors (e.g. INR correction, reversal agent use, and drug interruption) were associated with thromboembolism. Only one rebleeding event and no thromboembolic events occurred in patients without reversal agent use, heparin bridge, or anticoagulant interruption. Third, some endoscopic results and clinical outcomes differed between DOAC and warfarin users or between those with upper and lower GIB.

In agreement with our findings, Choudari et al[5] found no significant differences in endoscopy therapy need or rebleeding between anticoagulant users and non-anticoagulated users. Konstanticos et al[6] reported differences in transfusion need and mortality between anticoagulant users and non-matched controls with upper GIB. Our study also showed a similar rate of early endoscopy, detailed etiology of GIB, and endoscopy-related adverse events (0%) between the two groups, suggesting that early endoscopy can be safe for anticoagulated as well as non-anticoagulated GI bleeders.

The ASGE guideline recommends that INR < 2.5 is reasonable to perform endoscopic therapy[2]; however, the evidence for this is not well established. We found here that any INR category level—including INR ≥ 2.5—and a continuous INR value were not significant risk factors for rebleeding. Wolf et al[8] showed that neither a continuous INR value nor INR category was a predictor of rebleeding. Rubin et al[25] found that the rebleeding rate in patients with supratherapeutic INR (≥ 4.0) were not significantly different from those with INR 2.0–3.9. Taken together with our results, endoscopy in acute GIB would appear to be effective even in patients with elevated INR before the procedure. However, we found that INR ≥ 2.5 at onset was a significant predictor of thromboembolism. We believe this means that rapid correction of INR during the peri-endoscopic period in patients with INR ≥ 2.5 at onset confers increased risk. Because the ASGE guideline recommends performing endoscopic therapy in GI bleeding patients with INR < 2.5[2], many physicians quickly reduce the INR level in the peri-endoscopic period, especially in patients with INR ≥ 2.5 at onset. We also speculate that it takes several days for the INR to reach therapeutic anticoagulation[26], during which time a hypercoagulable state may occur due to the procedure itself or drug interruption[27], leading to thromboembolism risk.

No data are available on the role of heparin bridge in acute GIB, and endoscopic guidelines do not mention this topic[2,3]. In our study, heparin bridge did not significantly increase or decrease the risk of rebleeding, or thromboembolism. In a recent randomized controlled trial, the heparin bridge group experienced more major bleeding than the non-bridged group, with no difference in thromboembolism in the periprocedural period[28]. Therefore, heparin bridge might be ineffective in the acute GIB setting. We also had only one rebleeding event and no thromboembolic events in patients who had no reversal agent use, heparin bridge, or anticoagulant interruption. To summarize these points and the results of safe endoscopy in anticoagulant users, early endoscopy without INR correction, reversal agent use, heparin bridge, or anticoagulant interruption may be warranted for acute GI bleeders.

In our sub-analysis, DOAC users had a significantly higher rate of lower GIB and fewer received transfusions than warfarin users, and no significant differences were found between the groups in the rate of endoscopic therapy need, rebleeding, or thromboembolism. With respect to GIB outcomes, only one study (involving 13 dabigatran users and 26 warfarin users) has reported that dabigatran users had a lower rate of hypotension at baseline and a lower rate of transfusion need[13], similar to our findings. The reason for this remains to be elucidated, but warfarin users had a lower hemoglobin level and higher INR level at baseline, which affect blood loss, and this resulted in transfusion requirement.

Our study showed outcomes differences in anticoagulated patients between those with upper and lower GIB. Although 44% of patients used PPIs before the onset of upper GIB and all patients were administered them during their hospital stay, upper GI bleeders were associated with a higher rate of transfusion need and endoscopy therapy need than were lower GI bleeders. This finding suggests that anticoagulated GI bleeders may need to be managed differently, with upper GI bleeders handled more cautiously. Yamaguchi et al[29] showed that patients taking antithrombotics exhibited more severe clinical signs in upper GIB, which supports our findings.

One of the strengths of our study was the analysis of detailed clinical and endoscopic data that was collected from 314 GI bleeders. Another was that we identified a difference in the endoscopic results and clinical outcomes of the subgroup analyses of DOAC and warfarin users and of upper and lower GI bleeders. We also recognize several limitations. First, the 90-day outcome rate in anticoagulant users was 13.4% for rebleeding and 5.7% for thromboembolism in our study—similar to that of a Western study of 14%, and 4%, respectively[9]—but obtained with a relatively small number of subjects in this study. Second, INR correction, reversal agent use, and heparin bridging were at the discretion of the treating physicians, so further randomized controlled trials are needed to examine the role of these practices in managing acute GIB. Third, this was a single-center retrospective cohort investigation, and a prospective multicenter study is needed to generalize the results of this study.

In conclusion, endoscopy appears to be safe for anticoagulant users with acute GIB compared with non-users. Patient background factors were associated with rebleeding, whereas anticoagulant management factors (e.g. INR correction, reversal agent use, and drug interruption) were associated with thromboembolism. Early intervention without reversal agent use, heparin bridge, or anticoagulant interruption may be warranted for acute GIB.
